# Study of Fermentation Conditions Optimization for Xylanase Production by *Aspergillus tubingensis* FS7Y52 and Application in Agricultural Wastes Degradation

**DOI:** 10.3390/foods15020399

**Published:** 2026-01-22

**Authors:** Tianjiao Wang, Jinghao Ma, Yujun Zhong, Shaokang Liu, Wenqi Cui, Xiaoyan Liu, Guangsen Fan

**Affiliations:** 1 School of Food and Health, Beijing Technology and Business University, Beijing 100048, China; wangtianjiao1007@163.com (T.W.); 15032049018@163.com (J.M.); 18833106110@163.com (S.L.); 18232978688@163.com (W.C.); 2 Guangxi Key Laboratory of Agricultural Resources Chemistry and Biotechnology, Yulin 537000, China; arcb2020@163.com

**Keywords:** optimization, fermentation conditions, xylanase, *Aspergillus tubingensis*, agricultural waste degradation

## Abstract

This study aimed to systematically optimize the fermentation process for xylanase production by *Aspergillus tubingensis* FS7Y52, elucidate its enzymatic properties, and evaluate its application potential in the biodegradation of agricultural wastes. Key influencing factors were initially identified through single-factor experiments, followed by the screening of significant factors using the Plackett–Burman design. The optimal values were then approached employing the steepest ascent path method and Response Surface Methodology. The final determined optimal fermentation conditions were: corn husk (20–40 mesh) 40 g/L, tryptone 13.7 g/L, Tween-20 0.75 g/L, pH 6.5, fermentation temperature 42.1 °C, fermentation time 2 days, shaking speed 140 rpm, inoculum size 1 × 10^7^ spores/30 mL, and liquid loading volume 30 mL/250 mL. Under these conditions, xylanase activity reached 115.23 U/mL, representing a significant increase of 90.7% compared to pre-optimization levels. Studies on enzymatic properties revealed that the enzyme exhibited maximum activity at pH 5.0 and 55 °C, and demonstrated good stability within the pH range of 4.5–7.0 and at temperatures below 50 °C. In the degradation of agricultural waste, the enzyme system produced by this strain exhibits significant degradation effects on agricultural waste. A pronounced additive effect exists between xylanase and cellulase. When the dosages were 2430 U/g and 15.7 U/g for xylanase and cellulase, respectively, the maximum reducing sugar release reached 23.3%. The degradation rates of cellulose, hemicellulose, and lignin reached 57.8%, 51.9%, and 55.0%, respectively. Additionally, the strain itself exhibits significant degradation effects on substances such as cellulose in agricultural waste, achieving degradation rates of 78.8%, 70.8%, and 52.5% for cellulose, hemicellulose, and lignin, respectively. This study provides a solid theoretical foundation and technical support for the efficient production of xylanase by *A. tubingensis* and its industrial application in the resource utilization of agricultural wastes. From an economic perspective, the optimized strategy significantly enhances enzyme production efficiency while reducing substrate consumption and operational costs per unit of enzyme produced. This makes the resulting enzyme mixture more economically viable for large-scale applications. The utilization of this enzyme system to convert tobacco stems into sugars represents a compelling case for agricultural wastes reuse. It transforms residual biomass into high-value products, contributing to a circular bioeconomy by reducing waste and creating new renewable alternatives to conventional products. It provides an economically viable solution for the high-value utilization of woody lignocellulosic biomass.

## 1. Introduction

With the global emphasis on sustainable development and resource recycling, the efficient conversion and utilization of biomass resources have become a research hotspot [1,2]. Lignocellulose, as one of the most abundant renewable resources in nature, is primarily composed of cellulose, hemicellulose, and lignin [3,4]. Among these, hemicellulose is the second most abundant biopolymer after cellulose, mainly consisting of xylan, arabinan, mannan, etc. [5]. Xylan, the main component of hemicellulose, is a linear polymer composed of D-xylose units linked by β-1,4-glycosidic bonds [6]. Xylanase (EC 3.2.1.8) is a crucial class of enzymes that specifically hydrolyzes xylan into xylo-oligosaccharides and xylose. It holds significant application value in various fields such as food, feed, papermaking, and bioenergy [7,8,9]. Therefore, the development of highly efficient xylanases and research into their application in biomass degradation are of great importance.

Currently, xylanases are mainly derived from microorganisms such as bacteria, fungi, and actinomycetes [9,10]. Among these, fungi (e.g., *Aspergillus*, *Trichoderma*) are the primary production strains for industrial xylanase due to their high enzyme activity and stability [9,11,12]. However, significant differences exist in catalytic efficiency, pH stability, and thermotolerance among xylanases from different strains, making the screening of high-yield strains and optimization of their fermentation conditions crucial. *Aspergillus tubingensis*, a filamentous fungus widely distributed in nature, offers advantages such as rapid growth rate, strong adaptability, and ease of large-scale cultivation. It has been proven capable of producing cellulase–hemicellulase degrading enzyme mixtures [13,14].

However, the optimization of xylanase production in *A. tubingensis* remains limited. Most studies lack systematic optimization and economic evaluation, remaining at the “feasibility verification” stage. Their simplified experimental designs fail to thoroughly explore process optimization. For instance, Ristović et al. [15] conducted optimization experiments for xylanase production via solid-state fermentation, adding only a single type of agricultural waste as a carbon source to the basic medium and fermenting under constant temperature and humidity conditions to achieve maximum enzyme activity. However, they did not systematically optimize key process parameters such as fermentation duration. Similarly, Dos Santos et al. [16] also failed to optimize critical parameters like fermentation temperature and humidity. Furthermore, few studies systematically compare different agricultural wastes (e.g., wheat bran, rice husk, bagasse) under identical strains and cultivation conditions to determine the optimal substrate. For instance, Rastogi et al. [17] directly used corn cobs as the carbon source in liquid medium without exploring other agricultural wastes. Research on xylanase characterization derived from *A. tubingensis* remains limited. To date, only Intasit et al. [18] have conducted systematic purification and characterization of the enzyme, reporting an optimal pH of 5.0 and an optimal temperature of 50 °C. More importantly, the enzyme’s potential for practical biotechnological applications remains poorly understood and lacks experimental validation under industrially relevant conditions.

In the production process of xylanase, medium composition and fermentation conditions significantly impact enzyme activity. Traditional one-factor-at-a-time optimization methods are inadequate for comprehensively assessing the interactions among multiple factors. In contrast, statistical experimental design methods (e.g., Plackett–Burman (PB) design, Response Surface Methodology (RSM)) can efficiently screen key factors and determine optimal conditions [19,20]. Previous studies show that systematic optimization strategies can markedly enhance xylanase activity, providing a theoretical basis for industrial production. For instance, Dhaver et al. [21] optimized xylanase production conditions for *Trichoderma harzianum* using PB design and RSM, identifying key factors (e.g., pH, temperature, nitrogen source) and significantly increasing enzyme activity, demonstrating the effectiveness of statistical experimental design.

In practical applications, xylanase is often used in synergy with lignin-degrading enzymes such as cellulase to enhance the degradation efficiency of plant biomass and increase the release rate of reducing sugar from agricultural waste, thereby providing raw materials for the production of products like bioethanol and feed additives. For example, Kallel et al. reported that the synergistic treatment of cardboard waste with xylanase and cellulase significantly improved both the reducing sugar yield and bioethanol production [22]. Similarly, Yu et al. demonstrated that the combined application of xylanase and cellulase effectively enhanced the lignocellulose degradation efficiency of *Broussonetia papyrifera* [23]. China is the world’s largest producer and consumer of tobacco, but the tobacco production process generates approximately 1 million tons of waste materials such as tobacco stems each year [24]. As one of the primary agricultural wastes, tobacco stems are rich in cellulose and hemicellulose. The components of its cell wall account for approximately 35% to 40% of its dry weight. Excessively high cell wall substance content results in poor sensory quality, which is also a primary reason for its low utilization rate. Therefore, investigating the application of xylanase in tobacco stem degradation not only contributes to waste resource utilization but also provides technical support for the high-value utilization of other agricultural wastes.

To fully exploit the potential of *A. tubingensis* in xylanase production and deeply explore its application value in agricultural wastes resource utilization, this study focused on the optimization of fermentation conditions for xylanase production by *A. tubingensis*, the characterization of its enzymatic properties, and its application in agricultural wastes degradation. Through systematic optimization methods including single-factor experiments, PB design, steepest ascent path experiments, and RSM, this research aims to determine the optimal fermentation conditions for xylanase production by *A. tubingensis*, enhance enzyme activity, and provide a theoretical basis for industrial production. Concurrently, it seeks to comprehensively characterize the enzymatic properties of the xylanase, including its optimal pH and temperature and their stability, to clarify its applicability under different environmental conditions. Furthermore, the produced xylanase will be applied to tobacco stem degradation to investigate its effectiveness in the resource utilization of plant biomass. This work aimed to provide reliable technical support for the industrial production and application of xylanase from *A. tubingensis*, expand its application scope in areas such as bioenergy, food industry, and environmental protection, and contribute to the efficient conversion and sustainable development of agricultural wastes.

## 2. Material and Methods

### 2.1. Materials, Reagents, and Media Preparation

*A. tubingensis* FS7Y52 was isolated and preserved in our laboratory from the production environment of Baijiu (Chinese liquor) brewing. Beech xylan is sourced from Shanghai Yuanye Bio-Technology Co., Ltd. (Shanghai, China). CMC-Na was purchased from Beijing Mreda Technology Co., Ltd. (Beijing, China). Yeast extract powder, peptone, and tryptone are all sourced from Beijing Aoboxing Bio-Tech Co., Ltd. (Beijing, China). The product codes are: 01-012, 01-001, and 01-002. Tween-20 and Tween-40 are both supplied by Fuchen Chemical Reagents Co., Ltd. (Tianjin, China). Corn husks were collected in May 2024 from a neighborhood grocery store on Zengguang Road in Haidian District, Beijing, China. Hunan Tobacco Industry Co., Ltd. (Changsha, China) supplied the shredded tobacco stalks utilized in this study. Base fermentation medium for xylanase production was prepared with corn husk (20–40 mesh) 30.0 g/L, yeast extract powder 10.0 g/L, CaCl_2_ 0.3 g/L, KH_2_PO_4_ 0.6 g/L, MgSO_4_·7H_2_O 0.3 g/L, FeSO_4_·7H_2_O 0.3 g/L, natural pH (5.26, without adjustment). Fermentation medium for cellulase production was prepared with corn husk (40–60 mesh) 23.1 g/L, peptone 12.1 g/L, Tween-40 10.4 g/L, K_2_HPO_4_ 6 g/L, MgSO_4_ 2 g/L, CaCl_2_ 0.5 g/L, NaCl 2 g/L, pH 5.45. Fermentation medium from agricultural waste was prepared with tobacco stem (20–40 mesh) 40.0 g/L, tryptone 7.09 g/L, CaCl_2_ 0.3 g/L, KH_2_PO_4_ 0.6 g/L, MgSO_4_·7H_2_O 0.3 g/L, FeSO_4_·7H_2_O 0.3 g/L, Tween-20 0.75 g/L, pH 6.0. Yeast extract peptone dextrose medium (YPD) and potato dextrose agar medium (PDA) were prepared as previously reported [25,26,27]. All media were autoclaved at 121 °C for 20 min, and pH adjustment was performed only during media preparation. No further pH adjustments were made during fermentation.

### 2.2. Cultivation and Fermentation for Enzyme Production

*A. tubingensis* was inoculated onto PDA plates and incubated invertedly at 30 °C for 5 days. Spores were collected by rinsing the plates with 10 mL of sterile physiological saline. No wetting agents are used in the preparation of spore suspensions. The spore suspension was centrifuged at 5000 rpm for 5 min, after which the supernatant was removed. The pellet was resuspended in an equal volume of physiological saline, and spore counting was performed using a hemocytometer. The spore suspension was inoculated into the xylanase fermentation medium at a concentration of 1 × 10^7^ spores/30 mL and cultured at 30 °C with shaking at 180 rpm for 4 days. A 1 mL sample of the fermentation broth was centrifuged at 10,000 rpm for 10 min to obtain the crude enzyme solution [28].

### 2.3. Enzyme Activity Assay

#### 2.3.1. Xylanase Activity Assay 

**Figure A1:**
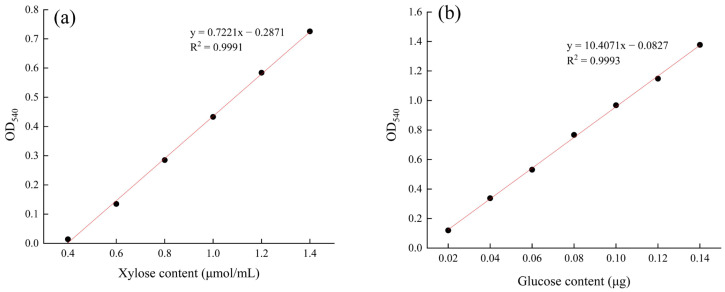
Standard curve. (**a**) Xylose standard curve. (**b**) Glucose standard curve.

A 25 μL aliquot of appropriately diluted enzyme solution (diluted with 0.05 mol/L pH 6.5 sodium disodium hydrogen phosphate–sodium dihydrogen phosphate buffer) was mixed with 225 μL of 1% beechwood xylan substrate and reacted at 50 °C for 10 min. Then 250 μL of 3,5-dinitrosalicylic acid (DNS) solution was added and mixed to terminate the reaction. After boiling for 15 min, the sample was cooled and 250 μL of 40% potassium sodium tartrate tetrahydrate solution was added to mix. The absorbance at 540 nm was then measured. Use the inactivated enzyme solution as a blank control. The amount of reducing sugar released was determined using the DNS method, with xylose as the standard. The xylose standard curve is shown in Figure A1a. One unit (U) of enzyme activity was defined as the amount of enzyme required to produce 1 μmol of xylose per minute [29].

#### 2.3.2. Cellulase Activity Assay 

A 50 μL aliquot of appropriately diluted enzyme solution (diluted with 0.05 mol/L pH 5.0 citric acid–sodium citrate buffer) was mixed with 150 μL of 1% sodium carboxymethyl cellulose (CMC-Na) and reacted at 50 °C for 30 min. Terminate the reaction using 200 μL of DNS solution, and after boiling, add 1 mL of deionized water to replace the potassium sodium tartrate tetrahydrate solution. Use the inactivated enzyme solution as a blank control. The amount of reducing sugar released was similarly determined using the DNS method, with glucose as the standard. The glucose standard curve is shown in Figure A1b. One unit (U) of enzyme activity was defined as the amount of enzyme required to produce 1 μmol of glucose per minute [30].

### 2.4. Optimization of Fermentation Conditions for Xylanase Activity

#### 2.4.1. Single-Factor Design

Based on the xylanase production fermentation medium, the following factors were optimized using single-factor design: type of carbon source, particle size of carbon source, carbon source concentration, type of nitrogen source, nitrogen source concentration, types of surfactant, surfactant concentration, initial pH, fermentation temperature, fermentation time, shaking speed, inoculum size and liquid loading volume (Table 1). All fermentations were performed in standard 250 mL erlenmeyer flasks without baffles. The initial liquid loading volume is 30 mL. Except for the specific optimization process for the liquid loading volume factor, particle loading and liquid loading volume remain constant at each test level in all other single-factor optimization experiments to ensure effective comparisons.

#### 2.4.2. PB Design

The PB design for eight variables (Table 2), including fermentation temperature (X_1_), tryptone concentration (X_2_), Tween-20 concentration (X_3_), initial pH (X_4_), shaking speed (X_5_), corn husk concentration (X_6_), inoculum size (X_7_) and fermentation time (X_8_) at two levels were used for screening, according to the results from the single-factor experiments. Each variable is represented at two levels, high and low, which are denoted by (+1) and (−1), respectively (Table 2). All experimental runs, comprising the 12-run PB design for screening eight continuous factors plus three center point runs (total n = 15 runs), were performed in a fully randomized order. Each set of experiments was repeated three times. The PB design and the response value of xylanase activity were shown in Table 2. A regression model was obtained based on the experimental data by Minitab software 17.1. The statistical significance was determined by *p*-value analysis, and the proportion of variance explained by the model obtained was given by the multiple coefficient of determination, R^2^.

Although the PB design is highly effective for rapidly screening key influencing factors in multivariate systems, it fails to account for potential interactions between factors. The variance component unexplained by linear models may be attributable to these overlooked synergistic or antagonistic effects. We addressed this limitation in subsequent work phases by adopting RSM, which specializes in modeling these quadratic and interaction effects, thereby providing a more comprehensive model of the system near its optimum points.

#### 2.4.3. Steepest Ascent Path Experiment Optimization

A steepest ascent path experiment was conducted for the significant factors identified by the PB design (direction determined by PB results, step size determined by single-factor results, PB outcomes, and empirical knowledge). The step sizes of the four significant factors are as follows: tryptone concentration 2 g/L, Tween-20 concentration 0.25 g/L, pH 0.5, fermentation temperature 3 °C. Based on the central value, a set of negative experiments and three sets of positive experiments were, respectively, set up. Each experimental group has three parallel runs. Non-significant factors were fixed at their optimal concentrations from single-factor optimization.

#### 2.4.4. Box–Behnken Experimental Design (BBD) Optimization

Using Minitab software, a three-factor, three-level BBD was employed to further investigate and optimize the factors identified as significant in the PB design. The center values were set based on the optimal combination from the steepest ascent path experiment. The design consisted of 12 factorial points, derived from the 2^2^ combinations for each pair of factors, augmented with three replicated center points. This resulted in a total of 15 experimental runs, with the inclusion of center points allowing for a robust estimation of pure error and for checking model curvature. All experiments were conducted in a completely randomized order. Each group of experiments was replicated three times.

### 2.5. Enzymatic Property Analysis

#### 2.5.1. Optimal pH and pH Stability

The xylanase activity was determined within the pH range of 2.0–10.0 to identify the optimal pH. Different buffers are used for different pH ranges, as follows: The 0.05 mol/L Gly-HCl buffer solution is used within the pH range of 2.0 to 3.5; For the pH range of 4.0–5.5, a 0.05 mol/L citric acid–sodium dihydrogen phosphate-buffer solution is used; The buffer solution used within the pH range of 6.0 to 8.5 is 0.05 mol/L sodium dihydrogen phosphate-disodium hydrogen phosphate solution; while for the pH range of 9.0–10.0, a 0.05 mol/L Gly-NaOH buffer is employed.

The enzyme activity at the optimal pH was defined as 100%. To evaluate pH stability, the enzyme solution was incubated with corresponding pH buffers at 25 °C for 30 min, followed by an ice bath. The residual enzyme activity was then measured, with the untreated enzyme solution serving as the control.

#### 2.5.2. Optimal Temperature and Thermal Stability

The optimal reaction temperature was determined by measuring enzyme activity within the range of 20–70 °C. To evaluate thermal stability, the enzyme solution was incubated at different temperatures for 30 min, and residual activity was measured under standard reaction conditions (55 °C, pH 5.0).

### 2.6. Agricultural Wastes Enzymatic Hydrolysis

#### 2.6.1. Enzyme Production by Fermentation

The crude xylanase solution was prepared under optimized fermentation conditions, and its activity was measured as described in Section 2.3.1. The cellulase production medium was prepared according to the formulation in Section 2.1, inoculated at 5% (spore count was 1 × 10^8^ spores/mL), and cultured at 31.5 °C and 180 rpm for 80.5 h. Cellulase activity was measured as described in Section 2.3.2.

#### 2.6.2. The Degradation of Agricultural Waste Culture Medium by the Strain

Prepare the tobacco stem fermentation medium according to the formula in Section 2.1. After preparing the spore suspension per the method in Section 2.2, inoculate the medium with 1 × 10^7^ spores. Incubate at 40 °C on a constant-temperature shaker at 140 rpm for 10 days. After fermentation, filter the medium through cheesecloth. Measure the reducing sugar content in 1 mL of the filtrate. Immediately place the filter residue in an oven to dry to constant weight. Collect it for determining cellulose, hemicellulose, and lignin content.

#### 2.6.3. Pretreatment of Agricultural Wastes

Exactly 10 g of cut tobacco stems were placed in a 2 L glass conical flask without a baffle plate and treated using a stepwise hot water extraction method: 1 L of distilled water preheated to 80 ± 0.5 °C was added. Soak for 1 h, stirring frequently during this period, then, an additional 1 L of distilled water at the same temperature was added, and extraction continued in a water bath at 80 ± 0.5 °C for 30 min. Solid and liquid phases were separated by suction filtration. The filtrate was discarded, and the residue was dried at 105 °C to constant weight to obtain the pretreated tobacco stem matrix, which was stored in a desiccator for further use. To investigate the mass conservation issue in the pretreatment process, we analyzed the moisture and ash content of the samples before and after pretreatment. The results indicate that, when evaluated based on consistent mass, pretreatment does not significantly alter the fundamental ash composition of biomass, thereby satisfying the mass balance principle for this key component.

#### 2.6.4. Enzymatic Hydrolysis Treatment

Accurately weigh 0.5 g of the pre-treated tobacco stems in the 50 mL glass conical flask without a baffle. Add the appropriate amount of enzyme solution based on the measured xylanase and cellulase activities (This section of the degradation experiment utilizes unpurified crude enzyme solutions.). Add 0.05 mol/L pH 5.0 citric acid–sodium citrate buffer to the reaction volume to a total of 15 mL, ensuring a solid-to-liquid ratio of 1:30. After sealing, enzymatic hydrolysis was carried out at 50 °C and 120 rpm for 5 h. A control sample with only buffer added was included. After hydrolysis, the mixture was filtered. A 1 mL aliquot of the filtrate was inactivated in a boiling water bath for 15 min for reducing sugar determination. The filter residue was dried and weighed for compositional analysis.

#### 2.6.5. Reducing Sugar Determination

The enzymatic hydrolysate was centrifuged at 10,000 rpm for 10 min, and the supernatant was appropriately diluted. A 250 μL aliquot of the diluted solution was mixed with an equal volume of DNS reagent, reacted in a boiling water bath for 15 min, cooled, and then 250 μL of 40% potassium sodium tartrate solution was added. After mixing, the absorbance was measured at 540 nm. Prepare a substrate blank control by conducting hydrolysis reactions under identical conditions using buffer solution instead of enzyme solution. These controls are subtracted from the corresponding sample readings. The reducing sugar content was calculated using a glucose standard curve (Figure A1b).

#### 2.6.6. Cellulose and Hemicellulose Determination

Exactly 0.3 ± 0.01 g of absolutely dry sample was placed in a stoppered test tube, and 3.00 ± 0.01 mL of 72% H_2_SO_4_ was added. Hydrolysis was performed in a water bath at 30 ± 3 °C for 60 ± 5 min, with occasional stirring. The mixture was transferred to a glass conical flask without a baffle and diluted with water to adjust the acid concentration to 4%. Hydrolysis was continued at 121 °C under high pressure for 1 h. After cooling and filtration, 10 mL of the hydrolysate was neutralized with calcium carbonate to pH 7, centrifuged, and filtered. Glucose (C-glu) and xylose (C-xyl) contents were determined by HPLC (Bio-Rad (Hercules, CA, USA) Aminex HPX-87H column, column and detector temperature 35 °C, flow rate 0.5 mL/min, mobile phase 5 mM H_2_SO_4_).

Cellulose content (%) = (C-glu × 86.73 × 0.90 × 10^−3^)/m_0_ × 100

Hemicellulose content (%) = (C-xyl × 86.73 × 0.88 × 10^−3^)/m_0_ × 100

C-glu is the concentration of glucose in the hydrolyzed sample solution, g/mL; C-xyl is the concentration of xylose in the hydrolyzed sample solution, g/mL; 86.73 is the volume of the hydrolysate filtrate, mL; m_0_ is the absolutely dry sample mass, g; 0.9 and 0.88 are conversion factors for glucan and xylan, respectively.

#### 2.6.7. Lignin Content Determination

Acid-insoluble lignin: The acid hydrolysis residue was washed with water to neutrality, dried at 105 °C to constant weight (M_1_), and then ashed at 575 ± 5 °C for 4 h. After cooling, the weight was recorded (M_2_).

Acid-insoluble lignin (%) = (M_1_ − M_2_)/m_0_ × 100

Acid-soluble lignin: After centrifuging the filtered hydrolysate at 10,000 rpm for 2 min, appropriately dilute the supernatant and measure the absorbance at 280 nm. The cuvette optical path length is 1 cm. (A, with deionized water as the blank; dilution was applied to ensure A between 0.7–1.0).

Acid-soluble lignin (%) = (A × D × V)/(23.35 × m_0_) × 100

D is the dilution factor, V is the hydrolysate volume 86.73 mL, 23.35 is the absorption coefficient, m_0_ is the absolutely dry sample mass.

### 2.7. Data Processing

All data are presented as the mean ± standard deviation of three replicates. All experiments in this study were conducted with biological replicates (independent flasks). Significance testing (*p* < 0.05) was performed using SPSS 24.0. Graphs were generated using Excel 2019 and Origin 2022.

## 3. Results and Discussion

### 3.1. Optimization of the Medium Composition by Single-Factor Design

**Figure 1:**
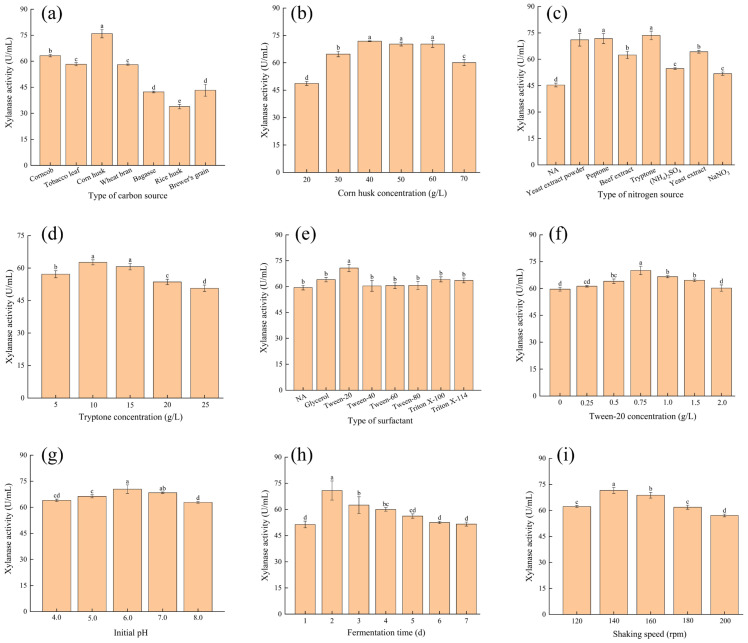
Single-factor optimization of the conditions for producing xylanase by *A. tubingensis*. (**a**) type of carbon source, (**b**) corn husk concentration (20, 30, 40, 50, 60, 70 g/L), (**c**) type of nitrogen source, (**d**) tryptone concentration (5, 10, 15, 20, 25 g/L), (**e**) type of surfactant, (**f**) Tween-20 concentration (0, 0.25, 0.5, 0.75, 1, 1.5, 2 g/L), (**g**) initial pH (4.0, 5.0, 6.0, 7.0, 8.0), (**h**) fermentation time (1, 2, 3, 4, 5, 6, 7 d), (**i**) shaking speed (120, 140, 160, 180, 200 rpm). Different letters above the bars indicate statistically significant differences at *p* < 0.05. “NA” indicates that no nitrogen source or surfactant was added.

**Figure A2:**
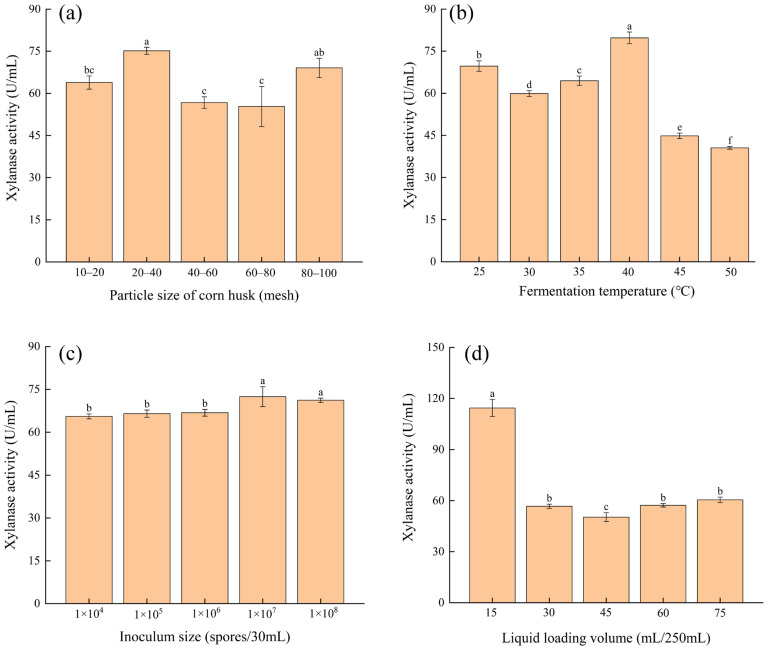
Single-factor optimization of the conditions for producing xylanase by *Aspergillus tubingensis* FS7Y52. (**a**) Particle size of corn husk (10–20, 20–40, 40–60, 60–80, 80–100 mesh). (**b**) Fermentation temperature (25, 30, 35, 40, 45, 50 °C). (**c**) Inoculum size (1 × 10^4^, 1 × 10^5^, 1 × 10^6^, 1 × 10^7^, 1 × 10^8^ spores/30 mL). (**d**) Liquid loading volume (15, 30, 45, 60, 75 mL/250 mL). Different letters above the bars indicate statistically significant differences at *p* < 0.05.

As shown in Figure 1 and Figure A2, seven factors, including type of carbon source, particle size of carbon source, carbon source concentration, type of nitrogen source, nitrogen source concentration, type of surfactant and surfactant concentration, were used to optimize the medium composition. The results of the carbon source optimization experiment (Figure 1a) showed that corn husk yielded the highest xylanase activity, reaching 75.97 U/mL, which was significantly higher than other carbon sources (*p* < 0.05). This is likely attributed to its richness in hemicellulose and porous fibrous structure. These characteristics not only effectively induce mold to secrete xylanase but also enhance solid–liquid contact efficiency, thereby promoting enzyme secretion and creating a favorable microenvironment for mycelial colonization and extracellular enzyme diffusion [31]. Corncob, tobacco leaf, and wheat bran showed moderate enzyme activities, indicating their potential as auxiliary carbon sources, though their inductive effects were less pronounced than that of corn husk. Compared to other agricultural residues, rice husk and tobacco leaf exhibit lower levels of cellulose and hemicellulose, while displaying significantly higher ash/silica content. This results in a relatively rigid and compact structure that impedes the effective enzymatic degradation of their hemicellulose. Similarly, wheat bran and corn cob, while holocellulose-rich, possess structural rigidity and may contain lignin or other components that slow initial microbial degradation. This could explain their lower performance as carbon sources for enzyme production compared to corn husk. The main component contents of various agricultural wastes are shown in Table A1. These results confirm that the synergistic effect of soluble inducers and insoluble matrices in lignocellulosic carbon sources may be a key factor driving high xylanase expression [8].

As shown in Figure A2a, the particle size of corn husk optimization experiment indicated that a mesh size of 20–40 resulted in the highest enzyme production, with an activity of 75.14 U/mL, significantly exceeding other particle sizes (*p* < 0.05). This suggests that an appropriate particle size provides an ideal pore structure and specific surface area, which facilitates microbial attachment and growth, as well as nutrient transfer and metabolic product diffusion. This particle size effect highlights the critical role of physical parameters in regulating microbial metabolism in solid-containing submerged fermentation systems. A moderate carbon source particle size optimizes triphasic (solid–liquid–gas) mass transfer efficiency, thereby significantly enhancing xylanase activity.

The corn husk concentration gradient experiment showed that xylanase activity reached its highest level at 40–60 g/L (*p* < 0.05) (Figure 1b). When the corn husk concentration was below 40 g/L, insufficient nutrient supply significantly restricted microbial growth and enzyme protein synthesis. In contrast, when the concentration exceeded 60 g/L, enzyme production markedly declined. This phenomenon may be attributed to multiple physiological inhibitory effects caused by high corn husk concentrations: when the carbon source concentration in the medium is excessively high, the osmotic pressure may increase, thereby affecting the normal metabolic activities of the strain. Meanwhile, excessive carbon sources accelerate microbial growth, which may also lead to dissolved oxygen limitations in the fermentation system, thereby interfering with enzyme synthesis and secretion. This concentration effect illustrates the dynamic balance between microbial growth and secondary metabolism. An appropriate carbon source supply meets the basic energy and carbon skeleton requirements for microbial growth while maintaining a favorable enzyme production microenvironment. Excessive concentrations disrupt this balance and inhibit enzyme biosynthesis through mechanisms such as osmotic stress and oxygen transfer limitation [32,33,34].

The nitrogen source optimization experiment revealed that peptone, yeast extract powder, and tryptone had significantly higher xylanase activities compared to other nitrogen sources (*p* < 0.05) (Figure 1c). These nitrogen sources were rich in amino acids and peptides, providing ample nitrogen and growth factors to promote microbial growth and metabolism. Additionally, they might activate nitrogen metabolic pathways within the strain, enhancing enzyme synthesis and secretion. Furthermore, these nitrogen sources could improve the strain’s antioxidant capacity and stress response, thereby enhancing its stability and enzyme production capability during fermentation [35,36]. In contrast, ammonium sulfate and sodium nitrate as sole nitrogen sources resulted in lower enzyme activity, likely due to their simpler nutritional profiles, which fail to meet the strain’s demand for diverse amino acids and growth factors.

The tryptone concentration gradient experiment (Figure 1d) indicated that xylanase activity reached its peak at 10–15 g/L (*p* < 0.05). When the tryptone concentration was below 10 g/L, limited nitrogen supply significantly restrained microbial growth and enzyme protein synthesis. Conversely, when the concentration exceeded 15 g/L, enzyme production decreased noticeably. This phenomenon may be due to multiple physiological inhibitory effects caused by high nitrogen source concentrations: firstly, high nitrogen levels may lead to elevated ion concentrations in the medium, affecting normal metabolic activity of the strain; secondly, high concentrations of nitrogen sources may have triggered a “nitrogen metabolite inhibition” effect, preferentially suppressing the synthesis of secondary metabolic enzyme systems, including xylanase; additionally, high nitrogen concentrations may alter the intracellular nitrogen metabolic balance, interfering with enzyme synthesis and secretion. This concentration effect underscores the dynamic balance between microbial growth and secondary metabolism. An appropriate nitrogen supply meets the basic nitrogen skeleton requirements for microbial growth while maintaining an optimal enzyme production microenvironment. Excessive concentrations disrupt this balance and inhibit enzyme biosynthesis through mechanisms such as ion stress and oxygen transfer limitation [6].

As shown in Figure 1e,f, the influence of different surfactant and concentrations on xylanase production demonstrated that Tween-20 had the most significant promoting effect compared to other surfactants. Xylanase activity was higher when Tween-20 concentration ranged between 0.5 and 1.5 g/L, with the peak activity observed at 0.75 g/L. It is hypothesized that at an appropriate concentration, as a surfactant, Tween-20 molecules can integrate into the lipid bilayer of microbial cell membranes, rendering the membrane structure more “loose” and thereby facilitating faster entry of nutrients (such as carbon and nitrogen sources) into the cells. At high concentrations, it may also form transient hydrophilic channels, leading to increased non-specific transmembrane transport of small molecules. Additionally, during fungal fermentation, Tween-20 interacts with hydrophobic components in the cell wall, “loosening” its dense structure and reducing physical resistance to the passage of macromolecules like proteins. In the extracellular environment, Tween-20 acts as a stabilizer, minimizing aggregation and inactivation of secreted proteins while maintaining enzyme activity in the fermentation broth. When the Tween-20 concentration was below 0.5 g/L, the reduction in interfacial tension was insufficient to fully exert its promoting effect. Conversely, when the concentration exceeded 1.5 g/L, excessive surfactant may exert toxic effects on the strain, inhibiting microbial growth and enzyme synthesis. Therefore, 0.75 g/L Tween-20 is identified as the optimal surfactant concentration for xylanase production by *A. tubingensis*.

### 3.2. Optimization of the Fermentation Conditions by Single-Factor Design

As shown in Figure 1g, within the pH range of 4.0 to 8.0, xylanase activity initially increased and then decreased, with the peaking around pH 6.0. By measuring the pH of the culture medium before and after fermentation, we found that when the initial pH of the medium was 5.26 prior to fermentation, the pH rose to 5.74 after fermentation. Therefore, we hypothesize that when the initial pH is adjusted to 6.0, the post-fermentation pH range will fall between 6.0 and 7.0. This allows the strain to maintain its optimal pH range for an extended period during fermentation, resulting in superior fermentation performance compared to the initial pH of 5.26. Relevant studies indicate that the optimal pH for most microbial fermentation-produced xylanases falls within the slightly acidic to neutral range. For instance, the optimal pH for xylanase produced by *Aspergillus niger* is 5.0 [37]. Punpaporn et al. [38] found that the optimal pH for the *Bacillus* RS3025 isolate is 7.0. Under suitable pH conditions, the active site of the enzyme and the binding site of the substrate are in an optimal ionized state, enabling efficient catalysis of the substrate hydrolysis reaction. In highly acidic or alkaline environments, enzyme activity significantly decreased. In excessively acidic conditions, an abundance of hydrogen ions (H^+^) competitively binds to the enzyme’s active site, interfering with the normal binding process between the enzyme and the substrate. This may also cause partial denaturation of the enzyme protein, leading to reduced activity. In highly alkaline environments, hydroxide ions (OH^−^) attack the acidic amino acid residues in the enzyme molecule, altering its charge distribution and spatial conformation, thereby disrupting the structure of the active site and impairing the enzyme’s catalytic function [39,40].

The impact of fermentation temperature on xylanase activity (Figure A2b) revealed that it reached its highest level at 40 °C. At this temperature, microbial metabolic activity was most vigorous, and the efficiency of enzyme synthesis and secretion was optimal. The rate of enzymatic reactions within the cells accelerated, and protein synthesis mechanisms operated efficiently, providing favorable conditions for the extensive synthesis of xylanase [41,42]. However, when the temperature was below 40 °C, microbial metabolic rates slowed, limiting enzyme synthesis. Conversely, when the temperature exceeded 40 °C, the high temperature caused denaturation and inactivation of the enzyme’s active site, while also disrupting intracellular protein structures and the integrity of biological membranes, inhibiting microbial growth and enzyme secretion. The optimal fermentation temperature for microbial xylanase production is closely related to the growth environment of the strain, typically ranging between 30–50 °C. For instance, Shikha et al. and Yasser et al. found that the optimal temperatures for xylanase production by *Bacillus safasii* and *Aspergillus niger* were 45 °C and 50 °C, respectively [43,44].

Results on fermentation time optimization (Figure 1h) showed that xylanase activity was highest on the second day. This phenomenon was closely related to the microbial growth cycle and the accumulation of metabolic products. During the initial fermentation stage (day 1), the microbial cells were in an adaptation phase with slow growth rates. As fermentation progressed, the microbial cells entered the logarithmic growth phase, with enhanced metabolic activity and a gradual increase in enzyme synthesis and secretion. By day 2, microbial growth and metabolism reached their optimal state, resulting in the highest xylanase activity. However, when fermentation extended beyond three days, the microbial cells entered the decline phase, with weakened metabolic activity and reduced enzyme synthesis and secretion. Additionally, prolonged exposure led to structural denaturation of xylanase and degradation by proteases synthesized by the strain itself, resulting in decreased enzyme activity.

As shown in Figure 1i, results from shaking speed optimization experiments indicated that 140 rpm was the optimal speed for xylanase. This phenomenon was closely related to the regulation of dissolved oxygen levels and mixing uniformity in the fermentation system. At 140 rpm, the dissolved oxygen levels and distribution of nutrients in the fermenter were most uniform, favoring microbial growth and metabolism. When shaking speeds exceed 160 rpm, excessive vibration can increase friction, potentially subjecting microorganisms to significant shear forces that may cause cell death. This can lead to reduced enzyme activity, deformation, and other issues in cells, ultimately affecting fermentation outcomes [32].

Results from inoculum size optimization experiments (Figure A2c) showed that xylanase activity was highest when the inoculum size ranged from 1 × 10^7^ to 1 × 10^8^ spores/30 mL. This phenomenon was closely related to microbial growth kinetics and metabolic balance. Within this inoculum range, microbial cells rapidly adapted to the fermentation environment and entered the logarithmic growth phase, where metabolic activity was most vigorous, and enzyme synthesis and secretion efficiency were optimal.

Liquid loading volume influenced microbial growth and enzyme activity by affecting dissolved oxygen levels and nutrient distribution. Adequate dissolved oxygen supports microbial respiration, promoting enzyme synthesis and secretion, while uniform nutrient distribution ensures efficient utilization of carbon and nitrogen sources in the medium. Results of the liquid loading volume optimization experiment (Figure A2d) showed that although xylanase activity peaked at 15 mL/250 mL liquid loading volume (114.35 U/mL, *p* < 0.05), the resulting culture contained minimal liquid post-fermentation. When the working volume is 15 mL, the total enzyme yield per bottle is 1715.2 U/flask. Although the volumetric activity was relatively low at 56.7 U/mL in a 30 mL working volume, the total enzyme yield per flask remained exceptionally high at 1700.9 U/flask. Although the enzyme activity per unit volume is high at a liquid loading volume of 15 mL/250 mL, the overall final enzyme activity does not differ significantly from that observed at a liquid loading volume of 30 mL/250 mL. Increasing the liquid loading volume reduces dissolved oxygen levels, limiting microbial growth and decreasing enzyme synthesis and secretion. Therefore, considering both the medium conditions and the need to avoid excessive loss of volumetric productivity, a working volume of 30 mL was selected as the optimal compromise. Subsequent experiments will utilize the results from single-factor experiments without further optimization.

### 3.3. Optimization of the Fermentation Conditions by PB Design

A PB design was employed to screen eight factors for significance (Table 2). The results revealed significant differences in xylanase activity under different factor combinations (41.4–88.4 U/mL). The highest enzyme activity (88.4 U/mL) was achieved in the center point run of Group 13, indicating that this region was close to the optimal response range.

**Figure A3:**
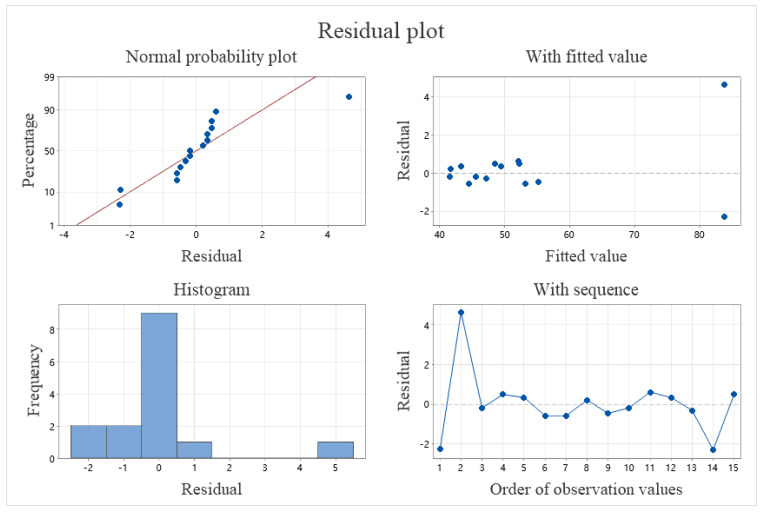
Residual plot for PB design (Normal probability plot, With fitted values, Histogram, With sequence). The red line in the normal probability plot represents the fitted straight line of the data. When the distribution of the values approximates a straight line, it indicates that the normality of the residuals is good.

Analysis of variance (ANOVA) (Table 3) showed that the model was significant, with an F-value of 53.94 and a *p*-value of 0.000 (indicating significance). Generally, a *p*-value less than 0.05 is considered statistically significant. The model diagnostics indicated robust performance: a high Adjusted R^2^ (0.9715) and a reasonable agreement with the Predicted R^2^ (0.9681) suggest the model has strong explanatory power and reliable predictive capability, rather than being overfit to the data. The responses at the three center points were 88.36, 81.44, and 81.42 U/mL. This low variability, with a standard deviation of approximately 4.0% relative to the mean, indicates good experimental reproducibility and consistency. Residual plot shown in Figure A3. The residuals in the figure show a random distribution, indicating that the model specification is reasonable. The identified key factors are robust and unaffected by systematic bias, making the regression results reliable for prediction and inference.

The PB design analysis was completed by calculating regression coefficients and statistical confidence levels. The regression equation for the activity of xylanase is as follows:

Xylanase activity = 25.1 − 0.0019 X_5_ − 0.0592 X_6_ − 0.000000 X_7_ + 0.449 X_1_ − 0.346 X_8_ + 0.432 X_2_ + 2.046 X_4_ − 8.52 X_3_

Among the eight variables examined, fermentation temperature (X_1_), tryptone concentration (X_2_), Tween-20 concentration (X_3_), and initial pH (X_4_) had significant effects on xylanase activity at the 5% significance level (Table 3). The regression coefficient for Tween-20 concentration (X_3_) was negative, while the coefficients for the other three variables were positive. Therefore, increasing Tween-20 concentration should be avoided in subsequent experiments.

### 3.4. Optimization of the Fermentation Conditions by Steepest Ascent PathExperiment

Based on the direction of significant factor effects determined by the PB experiment and the results of single-factor experiments, a steepest ascent pathexperiment was designed (Table 4). The fermentation temperature (X_1_) increment step size was set to +3 °C, the tryptone concentration (X_2_) increment step size to +2 g/L, the initial pH (X_4_) value to +0.5, and the Tween-20 concentration (X_3_) to −0.25 g/L. The enzyme activity initially increased and then decreased with the progression of experiments. The peak activity of 91.6 U/mL was achieved under the conditions of Run order 3 (fermentation temperature 43 °C, tryptone concentration 12 g/L, Tween-20 0.5 g/L, initial pH 6.5), representing a 3.5% improvement compared to the PB center point (88.4 U/mL). However, a further increase in factor levels (Run order 4) resulted in a decline in yield to 43.2 U/mL. This peak-and-decline profile indicates that Run order 3 lies at or near the maximum region of the response surface for this steepest ascent path. Accordingly, this parameter combination was selected as the center point for response surface optimization.

### 3.5. Optimization of the Fermentation Conditions by RSM Design

Based on the central values determined from the steepest ascent path experiment, this study selected three factors—fermentation temperature (A), tryptone concentration (B), and Tween-20 concentration (C)—for RSM experimental design. As shown in Table 5, xylanase activity varied significantly under different fermentation conditions: the lowest activity was observed in Test number 2 (43.1 U/mL), while the highest activity was achieved in Test number 12 (113.6 U/mL). To enhance experimental reliability, the center point experiments were repeated three times. Analysis of variance (F-test) indicated that the established quadratic model fitted the experimental data well. The coefficient of determination (R^2^) was 97.50%, indicating that the model explains 97.50% of the variation in the response values, with only approximately 2.50% of the total variation unexplained by the model. The estimated values, F-values, and *p*-values for each parameter are summarized in Table 6.

Through multiple regression analysis, the following quadratic polynomial regression equation for enzyme activity was derived (The regression uses coding units):

Xylanase activity = −7011 + 323.6 A + 53.0 B − 180 C − 3.797 A^2^ − 1.614 B^2^ + 79.1 C^2^ − 0.317 AB + 0.66 AC + 6.02 BC

**Figure A4:**
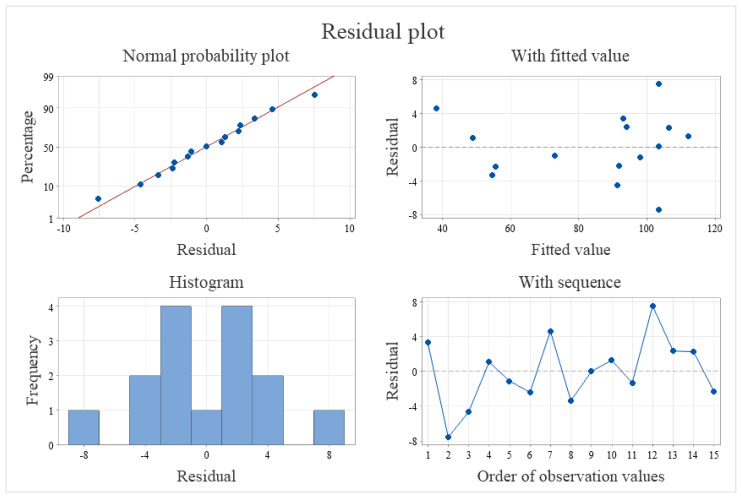
Residual plot for Response Surface Experiments (Normal probability plot, With fitted values, Histogram, With sequence). The red line in the normal probability plot represents the fitted straight line of the data. When the distribution of the values approximates a straight line, it indicates that the normality of the residuals is good.

The residual plot (Figure A4) was examined and showed a random scatter of residuals around zero, with no obvious patterns or trends. This, along with a satisfactory normal probability plot of the residuals, indicates that the underlying assumptions of the regression analysis (independence, homoscedasticity, and normality of errors) were reasonably met.

**Figure 2:**
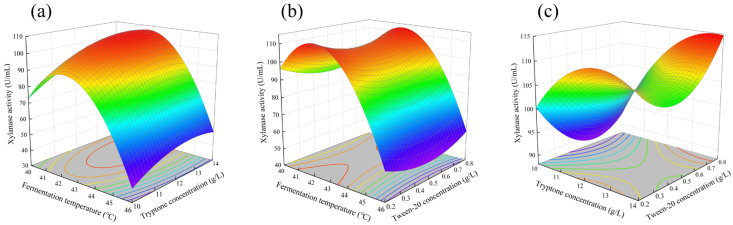
Three-dimensional surface maps of the effects of pairwise interaction of various factors on xylanase activity. (**a**) Interaction of fermentation temperature and tryptone concentration. (**b**) Interaction of fermentation temperature and Tween-20 concentration. (**c**) Interaction of tryptone concentration and Tween-20 concentration. Color gradient from blue to red indicates an increase in xylanase activity, where blue represents lower activity and red represents higher activity.

The response surface plot for xylanase activity corresponding to this model was shown in Figure 2. The response surface plots illustrated the interaction effects between any two variables by fixing the third variable at the zero level. Specifically, Figure 2a presented the three-dimensional response surface plot showing the effects of fermentation temperature (A) and tryptone concentration (B) on xylanase activity. It was observed that xylanase activity remained above 99.6 U/mL when the fermentation temperature was within 40.6–43.6 °C and the tryptone concentration was between 10.9–14.0 g/L. Figure 2b demonstrated the effects of fermentation temperature (A) and Tween-20 concentration (C) on xylanase activity. Fermentation temperature changes had a significantly greater impact on xylanase activity than Tween-20 concentration: an increase in fermentation temperature resulted in enzyme activity varying between 105.4–113.3 U/mL, whereas changes in Tween-20 concentration did not cause significant fluctuations in enzyme activity. Figure 2c showed the three-dimensional response surface plot of the effects of tryptone concentration (B) and Tween-20 concentration (C) on xylanase activity. Xylanase activity remained above 111.6 U/mL when the tryptone concentration was within 12.3–14.0 g/L and the Tween-20 concentration was between 0.7–0.8 g/L.

Using Minitab 19 software, the optimal values of each factor for maximizing xylanase activity were determined as follows: A (fermentation temperature) = 42.1 °C, B (tryptone concentration) = 13.7 g/L, and C (Tween-20 concentration) = 0.75 g/L. Under these conditions, the model predicted a maximum enzyme activity of 115.56 U/mL. To validate the reliability of the model prediction, verification experiments were conducted under the aforementioned optimal conditions. Independent biological validation of the optimized conditions (n = 3) yielded an average activity of 115.23 U/mL, with a 95% confidence interval of ±1.73 U/mL. This result closely matched the predicted value and represented a 90.7% increase compared to the pre-optimization level of 60.44 U/mL. Pre-fermentation conditions optimized as follows: corn husk (20–40 mesh) 30 g/L, yeast extract powder 10 g/L, fermentation temperature 30 °C, fermentation time 5 days, shaking speed 180 rpm, inoculum size 1 × 10^7^ spores/30 mL, and liquid loading volume 30 mL/250 mL.

In this study, key factors were screened through PB design, the maximum response region was approached via the steepest ascent path experiment, and RSM was employed to analyze nonlinear relationships between variables and achieve parameter optimization. Ultimately, xylanase activity was increased to 115.23 U/mL, significantly enhancing fermentation efficiency. The validation results were consistent with the model predictions, demonstrating that the established model was effective and reliable for optimizing and predicting the fermentation process of this enzyme.

Through PB experiments, response surface experiments, and other methods, we have achieved a certain degree of optimization in the fermentation process. The comparison of key parameters and performance metrics before and after system optimization is shown in Table A2. Our optimization conditions were compared with xylanase fermentation conditions reported for other *A. tubingensis* strains or similar fermentation models. The results obtained in this study demonstrate significant advantages in certain aspects. Tran et al. [45] found that the optimal pH for xylanase production by *Streptomyces thermocarboxydus* is 9.8; Ameen [46] observed that *Aspergillus fumigatus* strain KSA-2 exhibited peak xylanase activity at pH 9.0. Following optimization in this study, the fermentation medium pH reached 6.5—approaching neutral conditions. This simplifies pH adjustment steps, thereby reducing chemical costs associated with acids and bases while streamlining subsequent wastewater treatment processes. Compared to Layly et al.’s [47] findings (where *Bacillus halodurans* CM1 achieved optimal xylanase production at 200 rpm), strain FS7Y52 fermented at only 140 rpm, conserving energy and reducing equipment wear. Most studies indicate that fungi require extended periods for enzyme production. For instance, Salah et al. [48] reported that *Pichia membranifaciens* fermentation required 4 days to produce xylanase. However, this experiment achieved maximum enzyme activity after only 2 days of fermentation.

## 4. Enzymatic Properties

All enzyme preparations used in the enzymatic property determination experiments are crude enzyme solutions. The investigation of crude enzyme solution properties aligns with the downstream application of crude supernatant in this study (for biomass hydrolysis). However, it should be noted that other coexisting hydrolytic enzymes, such as other types of proteases, amylases, or lipases, may be present in the fermentation broth. These coexisting hydrolytic enzymes may influence the observed optimal pH, and thermal stability, potentially broadening or complicating the stability curve. Future research using purified enzymes remains necessary to elucidate the intrinsic characteristics of specific xylanases.

### 4.1. Optimal pH and pH Stability of Xylanase

**Figure 3:**
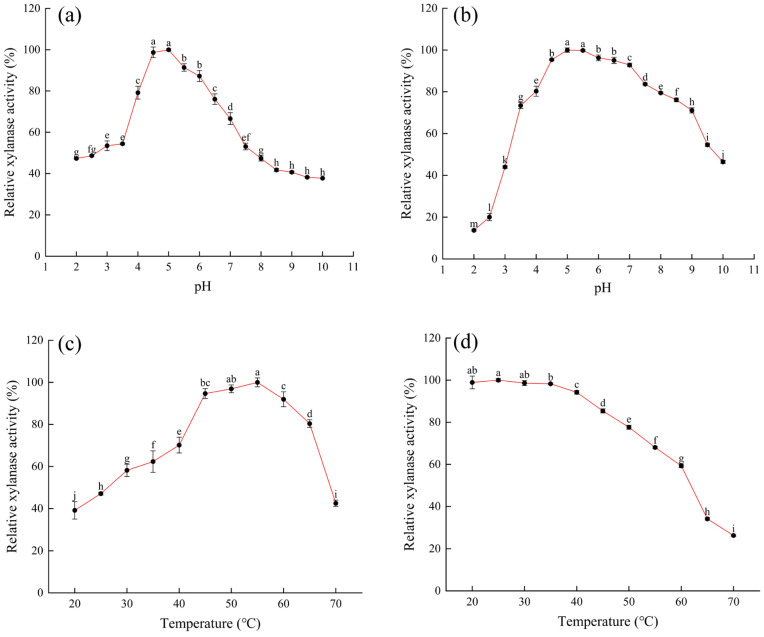
Enzymatic properties of the xylanase. (**a**) Optimal pH for xylanase (2.0, 2.5, 3.0, 3.5, 4.0, 4.5, 5.0, 5.5, 6.0, 6.5, 7.0, 7.5, 8.0, 8.5, 9.0, 9.5, 10.0). (**b**) Stability of xylanase under different pH conditions (2.0, 2.5, 3.0, 3.5, 4.0, 4.5, 5.0, 5.5, 6.0, 6.5, 7.0, 7.5, 8.0, 8.5, 9.0, 9.5, 10.0). (**c**) Optimum temperature for xylanase (20, 25, 30, 35, 40, 45, 50, 55, 60, 65, 70 °C). (**d**) Stability of xylanase under different temperature conditions (20, 25, 30, 35, 40, 45, 50, 55, 60, 65, 70 °C). Different letters indicate statistically significant differences at *p* < 0.05.

As shown in Figure 3a, the xylanase activity reached its peak at pH 5.0, indicating that this pH was the optimal condition for the enzyme’s catalytic function. From Figure 3b, it can be observed that the residual enzyme activity remained relatively high within the pH range of 4.5–7.0, with the highest residual activity occurring at pH 5.0. This suggested that the enzyme exhibited good stability and maintains high catalytic activity within this pH range. When the pH fell below 4.5 or exceeded 7.0, the enzyme activity showed a decreasing trend. At pH values below 2.5, the relative enzyme activity dropped sharply to below 20%. These results indicated that, in practical applications, the reaction environment should be maintained within pH 4.5–7.0 to ensure optimal catalytic efficiency of the xylanase. The optimal pH for enzyme activity (5.0) differs significantly from the optimal pH for strain growth and enzyme production during fermentation (6.5). A fermentation pH closer to neutral is more conducive to microbial health, efficient nutrient transport, and the functional operation of cellular secretion mechanisms, thereby maximizing enzyme yield. However, the enzyme’s own acidic optimum pH indicates its intended application or natural function occurs in weakly acidic environments.

Similar findings have been reported in other studies. For example, Xu et al. [49] described a xylanase from *Streptomyces* sp. FA1 with an optimal pH of 5.5 and high stability within the pH range of 3.0–11.0, which is consistent with the properties of the *A. tubingensis* xylanase described in this study. Research by Tran et al. [45] revealed that Xyn_TKU045 from *Streptomyces thermocarboxydus* exhibits optimal activity at pH 6 and demonstrates significant stability within the pH range of 6.0 to 8.0, inconsistent with the findings of this study.

### 4.2. Optimal Temperature and Thermal Stability of Xylanase

As shown in Figure 3c, the xylanase exhibited the highest activity at 55 °C. This indicated that, at this temperature, the enzyme molecules are in their most active state, with optimal collision frequency and binding probability between the active site and substrate molecules, thereby maximizing the catalytic reaction rate. Thermal stability test results (Figure 3d) showed that when the temperature was below 50 °C, the enzyme retained over 80% of its activity after 30 min of incubation, indicating good thermal stability within this temperature range. This is likely because, within this interval, thermal motion has not significantly disrupted the enzyme’s spatial conformation, allowing the active site to effectively bind with the substrate. However, when the temperature exceeded 50 °C, the residual enzyme activity gradually decreased. At 65 °C and above, the decline in enzyme activity became more pronounced, indicating severe denaturation and inactivation due to high temperature. These results suggested that, in practical applications, the enzyme should be prevented from exposure to high temperatures to maintain its stable catalytic activity. The research findings indicate that the xylanase produced by this strain exhibits average thermal stability, falling within the typical range for fungal xylanases. It does not demonstrate exceptional heat resistance. As reported by Lin et al. [50], the optimal temperature for xylanase XynST10 is 60 °C, while its enzymatic activity significantly decreases under both high-temperature and low-temperature conditions.

**Figure A5:**
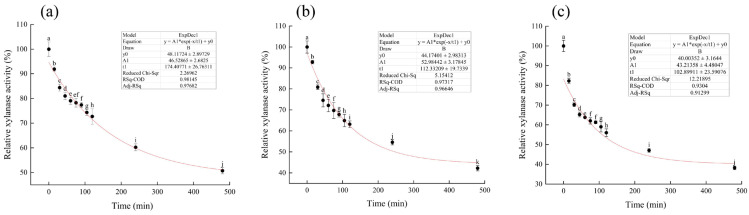
Half-lives of xylanase produced by *A. tubingensis* FS7Y52. (**a**) Half-life at 45 °C. (**b**) Half-life at 50 °C. (**c**) Half-life at 55 °C. Different letters indicate statistically significant differences at *p* < 0.05. The symbol “*” in the formula represents the multiplication sign.

To further investigate the stability of this enzyme, its half-lives (t½) were determined at 45, 50, and 55 °C, respectively. The results are shown in Figure A5. At these three temperatures, the enzyme’s half-lives were 559.4 min, 247.9 min, and 150.7 min, respectively. We observed that increasing the temperature from 50 °C to 55 °C (ΔT = 5 °C) resulted in a 97 min reduction in half-life, representing a 39% decay rate. This confirms that the enzyme’s stability significantly declines above 50 °C. Therefore, in practical operations, temperatures exceeding 50 °C should be avoided, as enzyme activity declines too rapidly at this point, making process stability difficult to control. We recommend an operational temperature window between 45 °C and 50 °C. Within this range, a reasonable balance can be achieved between maintaining high reaction rates and ensuring acceptable functional stability, thereby maximizing total product yield over time.

## 5. Hydrolysis of Agricultural Wastes

### 5.1. Analysis of Enzymatic Hydrolysis Efficiency

According to the results (Table 7), after treatment with different ratios of xylanase and cellulase, the contents of cellulose, hemicellulose, and lignin in tobacco stems significantly decreased. This indicated that both enzymes derived from *A. tubingensis* had a notable degrading effect on these major components. Below is the formula for calculating our degradation rate:

Degradation Rate (%) = [(C_initial − C_final)/C_initial] × 100

where C_initial represents the content (expressed in g per 100 g of dry raw biomass) of the specific component in the untreated, blank control sample.

C_final represents the corresponding content in the treated test sample.

All compositional analyses were performed on dried, milled solids. The percentage of all components (e.g., for cellulose, hemicellulose, and lignin) is calculated and reported based on initial dry weight. This is achieved by measuring the dry weight of solids before and after treatment, correcting the post-treatment component mass for total solids loss, and then expressing it as a percentage of the original untreated dry weight. This method ensures a clear mass balance closure and directly quantifies the proportion of original components dissolved or converted during processing, providing a clear indicator of process efficiency.

When xylanase and cellulase were added at 1500 U/g and 7.3 U/g, respectively, the degradation rates of cellulose, hemicellulose, and lignin were 21.8%, 28.3%, and 22.9%, respectively. When xylanase was added at 930 U/g and cellulase at 8.3 U/g, the cellulose degradation rate significantly increased to 54.4%, while the hemicellulose degradation rate decreased to 17.0%, and the lignin degradation rate was 42.7%. This indicates that cellulase exhibits high specificity for cellulose degradation, while simultaneously potentially reducing lignin content by disrupting the lignocellulosic matrix.

When the enzyme dosages were further increased to 2430 U/g of xylanase and 15.7 U/g of cellulase, the degradation rates of the three components reached 57.8%, 51.9%, and 55.0%, respectively, indicating a significant additive effect between the two enzymes. The mechanism may involve xylanase degrading hemicellulose and disrupting the cell wall network, making cellulose more accessible for degradation. Conversely, cellulase breaking down cellulose provides more sites for xylanase action, enhancing hemicellulose degradation. Due to the intense degradation of hemicellulose and cellulose, the lignin-carbohydrate complex (LCC) may dissociate, causing lignin fragments to partially dissolve into the reaction system and resulting in a decrease in lignin content. These findings aligned with recent studies, highlighting the importance of multi-enzyme additive effect in enzymatic hydrolysis systems.

In summary, the xylanase and cellulase produced by *A. tubingensis* were highly effective in degrading tobacco stems, particularly under additive conditions, significantly improving the degradation efficiency of the three components. This provided reliable technical support for the efficient conversion and utilization of other plant biomass and agricultural wastes.

### 5.2. Reducing Sugar Release of Enzymatic Hydrolysis

In terms of reducing sugar release, the blank control group without enzymatic hydrolysis showed a reducing sugar content of only 0.6%, indicating that carbohydrates in tobacco stems are difficult to naturally decompose into reducing sugars without enzymatic action. This low release rate was attributed to the complex structure of lignocellulosic biomass, where cellulose and hemicellulose were tightly encapsulated by lignin, limiting substrate accessibility [51]. When xylanase and cellulase were added at concentrations of 1500 U/g and 7.3 U/g, respectively, the reducing sugar content significantly increased to 7.0%, primarily due to the effective degradation of hemicellulose by xylanase, releasing xylo-oligosaccharides and xylose [52]. When xylanase and cellulase were added at concentrations of 930 U/g and 8.3 U/g, respectively, the reducing sugar content reached 17.5%, indicating high efficiency of cellulase in degrading cellulose and releasing more glucose and other reducing sugars [53]. When the dosages of xylanase and cellulase were increased to 2430 U/g and 15.7 U/g, respectively, the reducing sugar content further rose to 23.3%, confirming the strong synergistic effect between the two enzymes. This synergy not only improved the degradation efficiency of cellulose and hemicellulose but also promoted the substantial release of reducing sugars, providing abundant sugar substrates for subsequent bioconversion processes, such as fermentation for bioethanol production.

### 5.3. Degradation Effect of Agricultural Waste by A. tubingensis

When agricultural waste is used as the sole carbon source in the culture medium, fermentation for ten days after inoculation with spore suspension revealed that the strain exhibited a certain degree of degradation effect on agricultural wastes. The degradation results are shown in Table 8. In the reported data, the values for reducing sugars represent their concentration in the liquid fermentation broth, expressed as grams per liter (g/L) or as a percentage (*w*/*v*) of the liquid medium. In contrast, the contents of structural components such as cellulose, hemicellulose, and lignin are measured from the solid matrix and are reported as a weight percentage (wt%) relative to the initial dry mass of the solid substrate. After fermentation by the strain, the degradation rate of cellulose in tobacco stems reached 78.8%, with hemicellulose and lignin also showing varying degrees of degradation at rates of 70.8% and 52.5%, respectively. However, during growth, microorganisms actively absorb and utilize reducing sugars for biomass synthesis and energy metabolism—such as respiration and fermentation—to support growth and reproduction. Consequently, the reducing sugar content in the culture medium decreases rather than increases.

Therefore, the *A. tubingensis* strain exhibits significant degradation effects on agricultural waste, and the xylanase and cellulase produced by *A. tubingensis* effectively degraded cellulose, hemicellulose, and lignin in agricultural waste under synergistic conditions, demonstrating promising application potential. Future studies could focus on optimizing hydrolysis conditions, enhancing enzyme activity and stability, and exploring more efficient enzyme combinations and supplementary additives to further improve the degradation efficiency and resource utilization of agricultural wastes.

## 6. Conclusions

This study systematically investigated the medium composition and the fermentation conditions optimization, enzymatic properties, and agricultural wastes hydrolysis efficiency of xylanase produced by *A. tubingensis*. Through systematic optimization via single-factor experiments, PB design, steepest ascent path experiment, and RSM, the optimal parameters for xylanase production were determined as follows: corn husk (20–40 mesh) 40 g/L, tryptone 13.7 g/L, Tween-20 0.75 g/L, pH 6.5, fermentation temperature 42.1 °C, fermentation time 2 days, shaking speed 140 rpm, inoculum size 1 × 10^7^ spores/30 mL, and liquid loading volume 30 mL/250 mL. Under these conditions, the enzyme activity significantly increased to 115.23 U/mL, representing an improvement of 90.7% compared to the pre-optimization level. This fully demonstrated the effectiveness of the experimental design and optimization strategy. Enzymatic property analysis revealed that the xylanase exhibited the highest activity at pH 5.0 and 55 °C, with good stability within the pH range of 4.5–7.0 and at temperatures below 50 °C. These characteristics indicated that the xylanase maintained high catalytic efficiency in neutral to weakly acidic environments and under moderately high temperatures, suggesting potential industrial application value. The xylanase produced by this strain exhibits maximum catalytic activity at 55 °C. Meanwhile, the strain itself achieves peak enzyme production during fermentation at 42.1 °C. Selecting 42.1 °C maximizes the enzymatic reaction rate without significantly inhibiting microbial activity. In addition, our thermal stability analysis, quantified by half-lives measurements, indicates that prolonged exposure above 50 °C leads to rapid inactivation, which would severely compromise productivity in any extended process. Based on the above analysis, we recommend an operating temperature window of 45 ± 3 °C for this xylanase in industrial bioprocessing.

In the application study on enzymatic hydrolysis of agricultural wastes, the use of xylanase and cellulase at different ratios effectively degraded cellulose, hemicellulose, and lignin in tobacco stems. The optimal degradation efficiency was achieved when xylanase and cellulase were added at 2430 U/g and 15.7 U/g, respectively, resulting in a reducing sugar release of up to 23.3%, and degradation rates of 57.8%, 51.9%, and 55.0% for cellulose, hemicellulose, and lignin, respectively. Furthermore, analysis of the medium components before and after fermentation revealed that this strain also exhibits significant degradation effects on substances such as cellulose in agricultural waste, achieving degradation rates of 78.8%, 70.8%, and 52.5% for cellulose, hemicellulose, and lignin, respectively. These results not only validate the synergistic effect between the two enzymes but also provide technical support for the resource utilization of other plant biomass and agricultural wastes.

In summary, this study significantly enhanced the production of xylanase by *A. tubingensis* through optimization of medium composition and fermentation conditions, systematically characterized the enzymatic properties. The FS7Y52 crude enzyme solution obtained by this research demonstrates significant potential for direct application as an industrial-grade bioproduct in the biomass refining pretreatment stage. The operational profile of the enzyme system, specifically its broad pH tolerance (maintaining over 70% activity between pH 3.5–8.5) and its moderate thermal stability with an optimal functional window around 50–55 °C, aligns well with the common conditions of mild thermochemical or autohydrolysis pretreatment processes. Furthermore, the crude extract’s inherent multi-enzyme composition (primarily xylanase and cellulase activities) demonstrates a potent, additive capacity for deconstructing complex native lignocellulose. These properties collectively suggest that this minimally processed fermentation product could serve as a robust, cost-effective biocatalytic agent in biomass refining. Its stability under acidic to neutral conditions and at moderate temperatures may reduce the need for stringent pH or temperature control during application, thereby simplifying process design and improving overall process economics for the enzymatic valorization of agricultural residues. These findings lay a solid foundation for subsequent industrial production and application. Future research could further explore enzyme purification, immobilization, and application effects on different substrates to expand its potential uses in bioenergy, food industry, and environmental protection.

## Figures and Tables

**Table 1:** Factors and levels of single-factor design.

Factors	Levels
Type of carbon source	brewer’s grain, bagasse, corncob, corn husk, rice husk, wheat bran, tobacco leaf
Particle size of carbon source (mesh)	10–20, 20–40, 40–60, 60–80, 80–100
Carbon source concentration (g/L)	20, 30, 40, 50, 60, 70
Type of nitrogen source	NA, NaNO_3_, (NH_4_)_2_SO_4_, tryptone, peptone, yeast extract powder, yeast extract, beef extract
Nitrogen source concentration (g/L)	5, 10, 15, 20, 25
Type of surfactant	NA, Tween-20, Tween-40, Tween-60, Tween-80, Triton X-100, Triton X-114, Glycerol
Surfactant concentration (g/L)	0, 0.25, 0.5, 0.75, 1.0, 1.5, 2.0
Initial pH	4.0, 5.0, 6.0, 7.0, 8.0
Fermentation temperature (°C)	25, 30, 35, 40, 45, 50
Fermentation time (d)	1, 2, 3, 4, 5, 6, 7
Shaking speed (rpm)	120, 140, 160, 180, 200
Inoculum size (spores/30 mL)	1 × 10^4^, 1 × 10^5^, 1 × 10^6^, 1 × 10^7^, 1 × 10^8^
Liquid loading volume (mL/250 mL)	15, 30, 45, 60, 75

Note: “NA” indicates that no nitrogen source or surfactant was added.

**Table 2:** PB design matrix for evaluating factors influencing xylanase activity (n = 3).

Run Order	X_1_	X_2_	X_3_	X_4_	X_5_	X_6_	X_7_	X_8_	Xylanase Activity (U/mL)	CI 95%	
										Lower	Upper
1	35(−1)	5(−1)	1(+1)	7(+1)	160(+1)	30(−1)	1.5 × 10^7^(+1)	1(−1)	43.59 ± 1.68 ^gh^	39.43	47.75
2	45(+1)	5	1	5(−1)	160	50(+1)	0.5 × 10^7^(−1)	1	43.91 ± 0.28 ^gh^	43.21	44.61
3	35	5	0.5(−1)	5	120(−1)	50	1.5 × 10^7^	3(+1)	41.83 ± 0.37 ^hi^	40.93	42.74
4	45	15(+1)	0.5	5	160	30	1.5 × 10^7^	1	52.73 ± 0.56 ^cd^	51.34	54.12
5	45	5	0.5	7	160	50	0.5 × 10^7^	3	52.77 ± 1.21 ^cd^	49.77	55.78
6	35	15	1	5	160	50	1.5 × 10^7^	3	41.42 ± 0.28 ^i^	40.72	42.12
7	45	15	0.5	7	120	50	1.5 × 10^7^	1	54.76 ± 1.49 ^c^	51.05	58.47
8	45	5	1	7	120	30	1.5 × 10^7^	3	46.82 ± 0.97 ^f^	44.41	49.23
9	45	15	1	5	120	30	0.5 × 10^7^	3	49.73 ± 0.83 ^e^	47.67	51.79
10	35	15	0.5	7	160	30	0.5 × 10^7^	3	52.59 ± 1.66 ^d^	48.45	56.73
11	35	15	1	7	120	50	0.5 × 10^7^	1	48.99 ± 1.19 ^e^	46.03	51.95
12	35	5	0.5	5	120	30	0.5 × 10^7^	1	45.34 ± 0.21f ^g^	44.83	45.86
13	40	10	0.75	6	140	40	1 × 10^7^	2	88.36 ± 1.32 ^a^	85.08	91.65
14	40	10	0.75	6	140	40	1 × 10^7^	2	81.44 ± 1.18 ^b^	78.49	84.39
15	40	10	0.75	6	140	40	1 × 10^7^	2	81.42 ± 0.63 ^b^	79.86	82.97

Note: Different letters in the column of xylanase activity indicate statistically significant differences at *p* < 0.05.

**Table 3:** Statistical analysis in PB design for xylanase activity (n = 3).

Code	Variable	Low Level (−1)	High Level (+1)	df	Adj SS	Adj MS	Effect (EXi)	F- Value	*p*- Value	Rank	Significance
Model				9	3326.64	369.63		53.94	0.000		**
X_1_	Fermentation temperature (°C)	35	45	1	60.56	60.56	4.493	8.84	0.031	1	*
X_2_	Tryptone concentration (g/L)	5	15	1	56.08	56.08	4.324	8.18	0.035	2	*
X_3_	Tween-20 concentration (g/L)	0.5	1	1	54.49	54.49	−4.262	7.95	0.037	3	*
X_4_	Initial pH	5	7	1	50.25	50.25	4.093	7.33	0.042	4	*
X_5_	Shaking speed (rpm)	120	160	1	0.02	0.02	−0.077	0.00	0.961	8	
X_6_	Corn husk concentration (g/L)	30	50	1	4.21	4.21	−1.185	0.61	0.469	6	
X_7_	Inoculum size (spores/30 mL)	0.5 × 10^7^	1.5 × 10^7^	1	12.37	12.37	−2.031	1.81	0.237	5	
X_8_	Fermentation time (d)	1	3	1	1.44	1.44	−0.692	0.21	0.666	7	
Residual				5	34.26	6.85					
Lack of fit				3	2.20	0.73					
Pure error				2	32.07	16.03					
Cor total				14	3360.91						
	R^2^ = 0.9898, R^2^_adj_ = 0.9715, R^2^_pre_ = 0.9681										

Note: * *p* < 0.05, significant at 5% level; ** *p* < 0.01, significant at 1% level.

**Table 4:** Experimental designs and the results of steepest ascent path for xylanase activity (n = 3).

Run Order	Fermentation Temperature (°C)	Tryptone Concentration (g/L)	Tween-20 Concentration (g/L)	pH	Xylanase Activity (U/mL)	CI 95%	
						Lower	Upper
1	37	8	1	5.5	50.84 ± 0.55 ^c^	49.46	52.22
2	40	10	0.75	6	81.44 ± 0.73 ^b^	79.61	83.26
3	43	12	0.5	6.5	91.55 ± 0.73 ^a^	89.73	93.36
4	46	14	0.25	7	43.22 ± 0.55 ^e^	41.84	44.60
5	49	16	0	7.5	46.73 ± 0.62 ^d^	45.18	48.27

Note: Different letters in the column of xylanase activity indicate statistically significant differences at *p* < 0.05.

**Table 5:** BBD experimental design, response of the dependent variable, and its confidence interval (n = 3).

Test Number	Fermentation Temperature (°C)		Tryptone Concentration (g/L)		Tween-20 Concentration (g/L)		Xylanase Activity (U/mL)	CI 95%	
	A	Code A	B	Code B	C	Code C	Y	Lower	Upper
1	40	−1	10	−1	0.5	0	72.02 ± 0.86 ^g^	69.87	74.17
2	46	1	10	−1	0.5	0	43.08 ± 0.28 ^j^	42.38	43.78
3	40	−1	14	1	0.5	0	86.70 ± 0.91 ^f^	84.44	88.96
4	46	1	14	1	0.5	0	50.14 ± 0.37 ^i^	49.24	51.05
5	40	−1	12	0	0.25	−1	96.53 ± 2.54 ^d^	90.21	102.85
6	46	1	12	0	0.25	−1	51.39 ± 1.32 ^hi^	48.11	54.67
7	40	−1	12	0	0.75	1	96.39 ± 1.32 ^d^	93.12	99.68
8	46	1	12	0	0.75	1	53.24 ± 1.48 ^h^	49.55	56.91
9	43	0	10	−1	0.25	−1	96.67 ± 0.55 ^d^	95.30	98.04
10	43	0	14	1	0.25	−1	108.63 ± 1.59 ^b^	104.68	112.58
11	43	0	10	−1	0.75	1	89.56 ± 1.06 ^e^	86.93	92.20
12	43	0	14	1	0.75	1	113.61 ± 0.49 ^a^	112.40	114.82
13	43	0	12	0	0.5	0	96.07 ± 1.04 ^d^	93.49	98.66
14	43	0	12	0	0.5	0	111.16 ± 1.52 ^a^	107.39	114.94
15	43	0	12	0	0.5	0	103.59 ± 0.50 ^c^	102.36	104.84

Note: Different letters in the column of xylanase activity indicate statistically significant differences at *p* < 0.05.

**Table 6:** Regression coefficients and their significances for xylanase activity from the results of the BDD (n = 3).

Source	Degree of Freedom	Sum of Aquares	Mean Aquare	F-Value	*p*-Value	Significant
Model	9	8021.50	891.28	21.63	0.002	**
A: Fermentation temperature	1	2956.89	2956.89	71.75	0.000	**
B: Tryptone concentration	1	416.15	416.15	10.10	0.025	*
C: Tween-20 concentration	1	0.03	0.03	0.00	0.981	
A^2^	1	4312.37	4312.37	104.64	0.000	**
B^2^	1	153.92	153.92	3.73	0.111	
C^2^	1	90.28	90.28	2.19	0.199	
AB	1	14.50	14.50	0.35	0.579	
AC	1	0.98	0.98	0.02	0.883	
BC	1	36.29	36.29	0.88	0.391	
Residual	5	206.05	41.21			
Lack of fit	3	92.14	30.71	0.54	0.701	Not significant
Pure error	2	113.91	56.96			
Cor total	14	8227.56				
R^2^ = 0.9750, R^2^_adj_ = 0.9299						

* *p* < 0.05, significant at 5% level; ** *p* < 0.01, significant at 1% level.

**Table 7:** Efficiency of enzymatic hydrolysis of tobacco stems by *A. tubingensis* fermentation broth (n = 3).

Treatment	Reducing Sugar (%)	Cellulose (%)	Hemicellulose (%)	Acid-Soluble Lignin (%)	Acid-Insoluble Lignin (%)
Blank control	0.58 ± 0.01 ^d^	35.28 ± 1.53 ^a^	10.62 ± 0.42 ^a^	16.89 ± 0.09 ^a^	5.61 ± 0.14 ^a^
Xylanase 1500 U/g + Cellulase 7.3 U/g	7.04 ± 0.05 ^c^	27.56 ± 2.68 ^b^	7.59 ± 0.68 ^b^	12.44 ± 1.20 ^b^	4.91 ± 0.19 ^b^
Xylanase 930 U/g + Cellulase 8.3 U/g	17.51 ± 0.05 ^b^	16.06 ± 0.21 ^c^	8.82 ± 1.40 ^ab^	8.20 ± 0.14 ^c^	4.70 ± 0.20 ^b^
Xylanase 2430 U/g + Cellulase 15.7 U/g	23.31 ± 0.52 ^a^	14.91 ± 0.01 ^c^	5.13 ± 0.02 ^c^	6.20 ± 0.08 ^d^	3.92 ± 0.32 ^c^

The same letters in the same column indicate that the data do not differ significantly at 5% probability.

**Table 8:** Degradation Effectiveness of Agricultural Waste by *A. tubingensis* (n = 3).

	Cellulase Activities (U/mL)	Xylanase Activity (U/mL)	Reducing Sugars (%)	Cellulose (%)	Hemicellulose (%)	Acid-Soluble Lignin (%)	Acid-Insoluble Lignin (%)
Before fermentation	-	-	14.68 ± 0.18	26.85 ± 5.48	6.51 ± 0.18	9.36 ± 0.28	5.12 ± 0.20
After fermentation	81.66 ± 3.34	37.80 ± 0.40	8.03 ± 0.16	5.72 ± 0.15	1.89 ± 0.12	2.36 ± 0.02	4.51 ± 0.13

## Data Availability

The original contributions presented in this study are included in the article. Further inquiries can be directed to the corresponding authors.

## Appendix A

### Appendix A.3

**Table A1:** Cellulose, hemicellulose, lignin, and ash content in different agricultural wastes.

	Cellulose (%)	Hemicellulose (%)	Lignin (%)	Ash/Silica (%)
Bagasse	40–45	25–30	20–25	1–3
Corncob	35–40	35–40	15–20	1–2
Wheat bran	10–15	35–40	8–15	5–8
Corn husk	30–35	25–30	15–20	3–5
Tobacco leaf	20–30	15–25	15–25	10–15
Rice husk	30–35	20–25	15–20	15–20

### Appendix A.6

**Table A2:** Comparison of initial conditions and optimized conditions for each factor.

Factors	Initial Conditions	Optimized Conditions
Carbon source concentration (g/L)	30	40
Nitrogen source concentration (g/L)	7.09	13.7
Tween-20 concentration (g/L)	0	0.75
Initial pH	5.23	6.5
Fermentation temperature (°C)	30	42.1
Fermentation time (d)	5	2
Shaking speed (rpm)	180	140
Inoculum size (spores/30 mL)	1 × 10^7^	1 × 10^7^
Liquid loading volume (mL/250 mL)	30	30
Xylanase activity (U/mL)	60.44	115.23
